# The contribution of Portuguese nursing to universal health access and coverage

**DOI:** 10.1590/1518-8345.1068.2671

**Published:** 2016-03-04

**Authors:** Ananda Maria Fernandes, Aida Maria de Oliveira Cruz Mendes, Maria Neto da Cruz Leitão, Sérgio David Lourenço Gomes, António Fernando Salgueiro Amaral, Maria da Conceição Saraiva da Silva Costa Bento

**Affiliations:** 1 PhD, Professor, Escola Superior de Enfermagem de Coimbra, Coimbra, Portugal; 2 MSc, RN, Direção Geral da Saúde, Lisboa, Portugal; 3 Specialist, President, Escola Superior de Enfermagem de Coimbra, Coimbra, Portugal

**Keywords:** Nursing, Health Services Acessibility, Equity in Health, Health Systems

## Abstract

**Objective:**

to analyze the contribution of Portuguese nursing to improving universal health access and coverage by means of the identification of nurses in the health system; evolution of health indicators; and access-promoting systems, in which nurses play a relevant role.

**Method:**

this was documentary research of publications fromnational and international organizations on planning and health outcomes. Statistical databases and legislation on health reforms were consulted.

**Results:**

nurses represent 30.18% of human resources in the national health service; the systems of access promotion performed by nurses have good levels of efficacy (95.5%) and user satisfaction (99% completely satisfied); in the local care the creation of Community Care Units (185) occurred, and 85.80% of home consultations were performed by nurses.

**Conclusion:**

political strategies, the National Health Service and strengthening of human resourcesin healthcareare the main determinants. Nursing is the most numerous professional group in the National Health Service, however numbers remaindeficient in primary health care. The improvement of academic qualification and self-regulation of this professional group has allowed for better answers inimproving health for the Portuguese.

## Introduction

Over the past 40 years, the Portuguese society has undergone enormous political, economic and social transformations. The establishment of democracy, in Aprilof 1974, paved the way for the development of a country that was, after 48 years of dictatorship, lagging within Europe. To examine access, universality of health care and nurses' contributions tothat, the major changes in health resources in recent years, demographic evolution, professional nurses' training and organization are used as references.

### Health resources: from welfare to the National Health Service

Until 1974, the Portuguese had a welfare health care system, supported by social insurance, which funded access to care forthose to whomthe state considered itself a debtor. Only the indigent, attested by parish councils, were entitled to free care. Most of the population paid for poor quality of care in public hospitals, concentrated in three major cities (Lisbon, Porto and Coimbra), or the few private clinics that existed across the country, but which had higher incidence in these three cities. On the other hand, the need to offer epidemic-free harbors, thereby strengthening the economic weight that they had to trade and supply goods to an industrializing Europe, made the State assume the health authority as a control plan for the major epidemics. Also, with the creation - a pioneer in Europe - of a network of public health facilities which beganin 1971, but was not fully implemented in 1974, the shortage of health care provision was marked by a reductionist view of the determinants of health and disease, very focused on a biological or biomedical dimension, which was followed by an increase in medical specialties. The number of health professionals was likewise reduced. The provision of care was "a fragmented set of varied nature of health services - large state hospitals, an extensive network of Mercy hospitals, medical centers of the Social Welfare Medical Services; Public Health Services (health centers from 1971); municipal physicians; specialized services for maternal and child health, tuberculosis and psychiatric disorders; private sector particularly developed in the ambulatory area"^(1).^ The State contribution to health was not enough to account for 3% of the wealth produced in 1970^(^ 1 ^)^.

With democracy, the right of access to a universal, general and free health system (it leans toward being free of charge since the second constitutional revision of 1989) was consecrated in the Constitution of the Portuguese Republic of 1976 (Article 64), essentially provided by a public National Health Service (NHS), created in 1979. Currently, the system organization maintains a public component (National Health Service, NHS) and a contracted private component, making the State the main financier of healthcare in Portugal. In this system, the provision of care is ensured by a network of Primary Health Care (PHC), which is public (central pillar of health system), by a Hospital Care (HC) network, either public or private, and by a National Network of Continuing Integrated Care (NNCIC), created in 2006, which is essentially contracted. These three networks guarantee coverage in health promotion, disease prevention, treatment of acute situations, and management of chronic conditions.

### The Population

Portugal, in the mid-1970s, was a poor country with poorroad access to large centers, with an employed population, mainly in the primary (34.94%) and secondary (33.73%) sectors, and of low literacy(25.7% illiteracy). The infant mortality rate reached 37,900; deliveries were mainly performed at home, with poor care, by curious people (62.51% non-hospital care). Life expectancy was low (68.2 years)^(^ 2 ^)^. Overall, all socio-economic and health indicators were very unfavorable.

Presently, with a population of 10457.3 inhabitants, the Portuguese society grows older. If in 1970, the aging index was 34%, in 2013, it is 133.5% and the percentage of the population at a working age per elderly fell from 6.4 to 3.3. In 2013, births represented 39% of children born in 1960, with the synthetic fertility rate going from 3.2 to 1.21, one of the lowest in Europe. There is a reduction of young people under 15 years, 25.3% in 1981 to 14.7% in 2013. In the same period, there has been an increase in the elderly (over 65 years) from 11.5% to 19.6%^(^ 2 ^)^. 

These demographic changes pose new challenges to the health system and its professionals to better respond to the emerging needs.

### Nursing

With regard to nursing, there were about 3,000 nurses and 15,000 nurse aids in Portugal in 1974. The following year, educational institutions no longer offered courses for nurse aids; the course to promote nurse aids to nurses was encouraged; there was movementto a single level of training for access to the profession, the nursing program^(^ 3 ^)^.

In 1988, nursing education was integrated into the national education system (polytechnic subsystem), granting a bachelor's degree in three years. A decade later (1998), with Portugal's adherence to the Bologna process, the course became a four-year course, attributing nursing licentiate degrees. The first programs of master's degrees in nursing sciences started in 1991, and in the beginning of this millennium several master's courses started in different areas of specialization in nursing. This is also the date of the beginning of the first doctoral program in nursing (2001); there are three regular doctoral programs in nursing and health with a branch in nursing in Lisbon, Porto and Coimbra.

Rooted in this evolution, the Regulation of Nurses Professional Practice (RNPP) was published in 1996, which clarifies concepts, characterization of nursing care and specifies the competence of professionals legally qualified to provide them. The creation of the Order of Nurses (ON), in 1998, drove the development of the profession, promoting the defense of quality of care and giving nurses autonomy for the development, regulation, and control of the profession, thereby ensuring compliance with the ethics rules and code of conduct. The Code of Conduct (1998, 2009), part of the Order's Association, the Quality Standards of Nursing (2001), the definition of the competencies of the General Care Nurse (2003; 2011) and Specialist Nurses (2010; 2011) are structuring tools of the profession^(^ 4 ^)^.

Based on the above, the evolution of Portuguese Nursing has accompanied the changes occurringin the health system and society in general, thereby recovering a delay in training and professional practice.

Health changes resultfrommultiple determinants. Of these determinants, the macro-contextual and strategic ones are quite relevant, especially when a context switch occurs, such as that which differentiates the country before and after 1974. However, despite the significant increase in living standards and improved health in Portugal, regional and social class asymmetries remain evident. These inequalities have been recently deepened due to the economic crisis in the country and are reflected particularly in the access to health care and utilization of health resources^(^ 5 ^)^. Throughout this period, the overall improvement of living conditions strongly interacted with the performance of the national health system and human resources. It is difficult to distinguish the contribution of the different variables for improving care. Nevertheless, one can state that nurses are particularly relevant in areas such as health promotion, especially in maternal and child health, providing better access to care and community-based care.

The objective of this study was to analyze the contribution of Portuguese nursing to improving universal health access and coverage. Specifically, to identify the distribution of nurses in the health system; evolution of a set of health indicators after the creation of a universal access system; and access-promoting systems, in which nurses play a relevant role.

## Method

A qualitative methodology was used, consisting of content analysis of a documentary *corpus* of documents fromnational and international organizations on health planning and outcomes, namely the General Direction for Health (GDH), Central Administration of the Health System (CAHS) and Organization for Economic Cooperation and Development (OECD). Statistical databases of the National Statistics Institute (NSI), PORDATA and the World Health Organization (WHO), as well as legislation on health reforms, were consulted. To analyze the contribution of Portuguese nursing to improving access and universal coverage in health, five different types of indicators were used: 1) human resources: number of nurses and their geographical distribution per work area; 2) performance of nurses in particularly sensitive areas to improve access to health care: line 24 and home visits; 3) results of epidemiological and preventive surveillance: vaccination coverage rate; 4) improvement of the health status of the Portuguese: infant and maternal mortality rate; and, 5) organization and management of care units: number of Care Units in the Community.

## Results

### Nurses in the Health System

The provision of nursing care in Portugalgrew as the global supply of care increased. By comparing the numbers of nurses in the health system, it is found that it almost doubled between the 1970s and the 1980s, following the growth in the number of hospital beds. In 2012, there were 39,797 nurses workingin the system. However, this number decreased by about 2% in 2013,to 38,937^(^ 6 ^)^.

Despite the increasing orientation of health policies for primary health care, of the 66,452 nurses enrolled in the Order of Nurses in 2015, only 11.51% worked in this area, while 51.46% worked in hospitals. The lowest percentage (0.61%) of those who worked in liberal regime should be noted^(^ 7 ^)^. 

These figures represent a ratio of 3.54 nurses per 1,000 people, one nurse for every 1.57 physicians^(^ 8 ^)^. In Primary Health Care, that rate is even lower than the national average (1.05), and less than one in two regions of the country, which is higher the concentration of hospital services (central region, Lisbon and Vale do Tejo)^(^ 8 ^)^.

### The contribution of nurses in access-promoting systems

The National Health Plan 2012-2016, in its strategic axis "Equity and access to health care", lists four systems dedicated to facilitating and defining access priorities: health line 24, Manchester triage priority system in the emergency room, green way toacute myocardial infarction, stroke and, lastly, green way to sepsis^(^ 9 ^)^. The participation of nurses is relevant in two of these four systems - health line 24 and Manchester's triage prioritysystem in the emergency room - since its implementation relies solely on nurses' actions.

The Service Center of the National Health Service, called health line 24, is a multi-channel service (telephone, web, e-mail and fax) that operates 24 hours a day, with nationwide coverage, performing triage, counseling and referral of ill citizens, including urgent problems, thus facilitating access to health services more rationally and responding to the needs regarding health expressed by citizens^(6).^ This care is performed exclusively by nurses, who advise, refer or help each citizen seeking this service resolve the situation alone, thereby reducing the use of hospital emergency departments. It was established in 2007 and currently incorporates the health care services of pediatrics 24 and public health line, a service focused on the user, inserted in the chain of health care provision and located in the system entry point.

From 2007-2013, there was positive growth in the demand for this service (10), as shown in Table 1.

**Table 1:** - Distribution of contacts and health line 24 efficacy rate. Portugal, 2015

**Year**	2007*	**2008**	**2009**	**2010**	**2011**	**2012**	**2013**
Total received contacts	298190	499342	1604477	650731	680533	798384	720897
Total attended contacts	295247	471510	1297685	621212	648435	763491	691367
Efficacy	99.0%	94.4%	80.9%	95.5%	95.3%	95.6%	95.9%
Mean contacts per day	1167	1292	3555	1702	1777	2086	1894

* From April 25 to December 31

**Figure 1:**
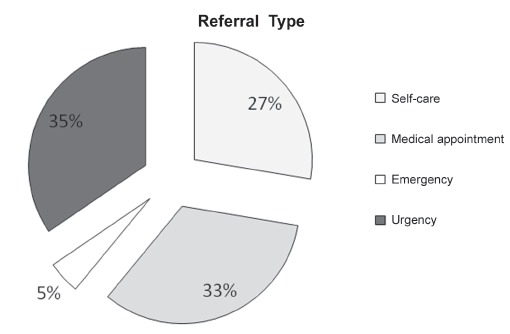
- Distribution of the type of referral performed by health line 24, Portugal, 2013

In 2013, 718,572 phone calls were received, of which more than 95% (689,042) were fulfilled, representing a mean of 2,057 missed calls a day. Of the received contacts, 75% are classified as triage, counseling and referral, providing a clinical customer service, assessing the level of risk based on the symptoms described by the user with counseling, including self-treatment and, if necessary, referring the patient to an institution in the network to provide more appropriate care. Nurses' assessment resulted in fewer than half of cases referred to urgent/emergency care services (40%), about 1/3 of calls being referred to medical appointments, and over 27% of cases resolved with advice, and with no other other contact with health services being necessary(6), as shown in Figure 1.

In the population group over 65 years, over 36% of users werereferred to a hospital emergency service due to frailty in 2013.

This service provided by nurses improves not only access but the efficiency of service usage. In the analysis of user referrals in 2013, it was found that over 50% of users who called planned to go to an emergency room, however they were referred to primary health care (30.2%) or self-care (24.7%). On the other hand, nearly 30% of users calling with the intention to monitor their health at home were referred to an urgent/emergency service^(^ 6 ^)^.

A study performed between May 1 and July 31, 2014^(^ 11 ^)^ showed that of the 51% of users that planned to seek an emergency service, 50.5% did not, because they had their situation solved with self-care or care primary health.

Thus, satisfaction with the service as examined by an independent entity (Nielsen, Portugal), is always greater than 95%, as shown in Table 2.

**Table 2:** - Degree of customer satisfaction with the service health line 24 (2009-2013). Portugal, 2014

**Satisfaction/Year**	**2007**	**2008**	**2009**	**2010**	**2011**	**2012**	**2013**
Highly satisfied	No data	No data	97%	98%	98%	99%	99%

### Nursing home visits 

Home visits are an important strategy for community-based care and for improving access and universality of care. Home care is a consultation provided by a health professional at home, in nursing homes or similar institutions, consisting of scheduled or unscheduled episodes, addressed to a user^(12)^.

The data show that most of these appointments are performed by nurses (Table 3), and their number has been growing^(^ 13 ^)^.

**Table 3:** - Registered home visits by a health professional. Portugal, DGS, 2014

**Home Visits**	**Absolute number (2011)**
Physicians	196769
Nursing	2115312
Social service	32562
Others	120846
Total	2465489

### Vaccination - results from the national program

**Figure 2:**
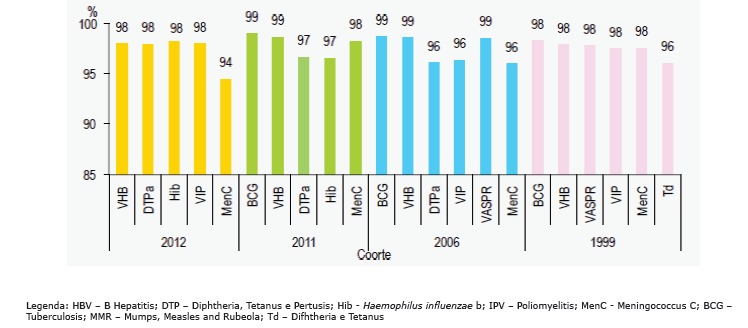
- Assessment of PNV coverage by cohort and vaccine. Portugal, 2104

The National Immunization Program (NIP) started in October of 1965^(^ 14 ^)^. It is universal, free to the user, decentralized, nationally managed, taken as a universal recipe, although it not mandatory, and it is essentially implemented by nurses of the public primary health care. Assessment of compliance with the National Immunization Program (NIP), which takes place on an annual basis, has allowed determining vaccination coverage rates at key ages. Overall, they reach adequate levels to confer immunity to different groups, with international requirements and commitments being fulfilled^(^ 14 ^)^, as shown in Figure 2.

### The evolution of health indicators

The investment in vast multidisciplinary teams that work in liaison with the primary and hospital level services is considered relevant in maternal and child health, a key factor for the progress of the indicators in this area. The inclusion of general care nurses and, particularly, of nurses specialized in maternal health, obstetric and child/pediatric health was considered mandatory in the various institutions of the reference network and functional coordinating units.

The infant and maternal mortality rates have decreased considerably, following the political and social changes in the country. Regarding the infant mortality rate, there has been significant progress, from 55.5 cases per 1,000 inhabitants (1970) to 2.8(2014). The maternal mortality rate also showed a considerable decrease. In 1970, it was 73.4 per 1 million, dropping to 19.0 in 1980. Beginning in1992, it always had values lower than 10 per one million, and in 2013, it was lower than six^(^ 2 ^)^.

### The organization and management of care units: Care Units in the Community

The Care Units in the Community (CUC) were established in 2008 to reform primary health care and have a mission to contribute to improving the health status of the population of the geographical area of intervention. They provide health care, psychological, social, home and community support, especially to individuals, families and vulnerable groups in higher risk situations, physical and functional dependence or illness that requires close monitoring. They also act in health education, integration into support networks forfamily, and implementation of mobile intervention units. These units are formed by independent professional teams, designated by Community Intervention Teams (CIT) to approach groups/communities in different contexts and according to the National Health Plan, by Continuous Care Teams (CCT) and by Community Support Teams in Palliative Care (CSTPC), to intervene with the elderly and/or the dependent. The coordinator of the CCT is designated among nurses with at least the nurse specialist training and experience in the functional area. According to the latest data, there are currently 185 operating CCTs^(^ 15 ^)^.

## Discussion

It was found that nurses working in the three care networks that compose the public and private provision, namely: primary health care network, with the Groups of Health Centers, Family Health Units and others functional units, where the Community Care Units (CCU)are highlighted, managed by nurses; special care network, with hospitals; integrated continued care network, with the units of care for convalescence, medium and long-term care and palliative care. In addition to these networks, it is possible to find nurses working in other care settings such as nursing homes, although only a few.

The distribution of nurses throughout all these units has guaranteed access to care, facilitating access to essential care. However, the ratio of nurses per inhabitants (3.54/‰) is wellbelow the mean ratio of OECD countries. Additionally, the nurse/physician rate (1.5) is also below the mean of countries in that organization (2.8)^(^ 16 ^)^. This nurse/physician rate can indicate an inefficient management of resources and potential nurses' skills in the care system^(^ 17 ^)^. The situation is even more severe in the primary care context, due to the low recruitment, showing a clear devaluation of the nurses' role in the local care of citizens. Nevertheless, the number of home visits and, in particular, its proportion to the performance of other health professionals is quite relevant. In fact, most of the care required by people when they are ill in their homes is nursing care. However, thesmall number of functioning CCUs is still noted. These are an organizational option that allows nurses to develop care strategies focused on the needs of the people in the exercise of their autonomy and in collaborative multidisciplinary teams.

On the other hand, the Portuguese nurses have given a significant contribution to accessibility and more rational use of the system. An example of this type of intervention is the implementation of health service line 24, which has allowed for an improvement of appropriate access to different health care with a high degree of satisfaction.

The NIP, with a mean vaccination coverage rate of 98% for each type of vaccine in the plan, nationally and internationally recognized, was and is an efficient and effective program that allowed for the eradication, elimination and control of the target diseases, contributing decisively to reducing child mortality and morbity-mortality from vaccine-preventable diseases, such as tuberculosis. The success was and is a combination of several factors, highlighting the commitment and acceptance of health professionals, among which nurses stand out as first-line professionals. Their proximity and accessibility to the entire population in Health Centers and its extension enables the management of records, notifying, informing and motivating citizens to maintain the vaccine schedules of the whole population. The massawareness and vaccination campaigns of children and adolescents in schools of school health programs have greatly contributed to these results^(^ 6 ^)^.

In 1970, all health indicators and, in particular, maternal and child indicators, assumed values of underdeveloped countries. The creation of national universal health care service, which is general and tends toward being free of charge, with greater supply of local care, associated with various social policies and measures such as mandatory inclusion of primary care nurses and, specifically, nurse specialists in maternal, obstetric and child health, in the different institutions of the referral network and functional units coordinators, were important to improving health indicators.

Investment in vast multidisciplinary teams that work in liaison with the primary and hospital level services is considered relevant in maternal and child health, consisting ofa determining factor in the progress of the indicators in this area.

## Conclusion

The creation of a universal health care system has enabled health services to be broughtcloser to the people, having gradually increased the centrality on primary health care and improved densification and articulation between the various levels and care units. The school and maternal health campaigns, with encouragement forassisted delivered at maternity hospitals, has decreased the maternal and infant mortality indicators to levels that are lower than in some Western European countries. The nursing workforce was increased and qualified, assuming new roles and effective responses to the health needs of the population. However, significant reinforcements are still required in primary health care, especially nurses with specialized graduate training and more organizational units coordinated by nurses.
